# Mechanical Assist Device-Assisted Percutaneous Coronary Intervention: The Use of Impella Versus Extracorporeal Membrane Oxygenation as an Emerging Frontier in Revascularization in Cardiogenic Shock

**DOI:** 10.7759/cureus.33372

**Published:** 2023-01-04

**Authors:** Neel Vora, Rajvi Chaudhary, Hetarth Vivek Upadhyay, Ashwati Konat, Parit Zalavadia, Arif Padaniya, Parth Patel, Nihar Patel, Priyadarshi Prajjwal, Kamal Sharma

**Affiliations:** 1 Department of Internal Medicine, B.J. Medical College, Ahmedabad, IND; 2 Department of Zoology, Biomedical Technology, and Human Genetics, Gujarat University, Ahmedabad, IND; 3 Department of Internal Medicine, GCS (Gujarat Cancer Society) Medical College, Ahmedabad, IND; 4 Department of Internal Medicine, Pramukhswami Medical College, Karamsad, IND; 5 Department of Medicine, Bharati Vidyapeeth University Medical College, Pune, IND; 6 Department of Cardiology, U.N. Mehta Institute of Cardiology & Research Centre, Ahmedabad, IND

**Keywords:** ventricular assist devices, cardiogenic shock, revascularization, ecmo, impella

## Abstract

The extracorporeal membrane oxygenation (ECMO) procedure aids in the provision of prolonged cardiopulmonary support, whereas the Impella device (Abiomed, Danvers, MA) is a ventricular assist device that maintains circulation by pumping blood into the aorta from the left ventricle. Blood is circulated in parallel with the heart by Impella. It draws blood straight into the aorta from the left ventricle, hence preserving the physiological flow. ECMO bypasses the left atrium and the left ventricle, and the end consequence is a non-physiological flow. In this article, we conducted a detailed analysis of various publications in the literature and examined various modalities pertaining to the use of ECMO and Impella for cardiogenic shocks, such as efficacy, clinical outcomes, cost-effectiveness, device-related complications, and limitations. The Impella completely unloads the left ventricle, thereby significantly reducing the effort of the heart. Comparatively, ECMO only stabilizes a patient with cardiogenic shock for a short stretch of time and does not lessen the efforts of the left ventricle ("unload" it). In the acute setting, both devices reduced left ventricular end-diastolic pressure and provided adequate hemodynamic support. By comparing patients on Impella to those receiving ECMO, it was found that patients on Impella were associated with better clinical results, quicker recovery, limited complications, and reduced healthcare costs; however, there is a lack of conclusive studies performed demonstrating the reduction in long-term mortality rates. Considering the effectiveness of given modalities and taking into account the various studies described in the literature, Impella has reported better clinical outcomes although more clinical trials are needed for establishing the effectiveness of these interventional approaches in revascularization in cardiogenic shock.

## Introduction and background

Acute cardiogenic shock (CS), which is a state of end-organ failure, occurs because of insufficient cardiac output as a result of ventricular dysfunction [1]. Clinically, CS is a state of hypoperfusion of organs and tissues, which manifests as a result of cardiac failure [2]. This is characterized by persistent hypotension, reduction in cardiac index, raised cardiac filling pressures, and pulmonary capillary wedge pressure (PCWP), along with clinical features of hypoperfusion (altered sensorium, cool extremities, and reduction in urine output) [1,3-4].

Despite improvements in treatment options, CS is a usual cause of mortality and management is still difficult. Impaired cardiac performance leading to hypoperfusion of organs, reduction in cardiac output, and hypoxia are the causes of CS [5]. Of CS patients, 81% have an acute myocardial infarction (AMI) [6].

Patients with ST-segment elevation myocardial infarction (STEMI) are at a greater risk of developing CS (around two times greater risk) compared to patients facing non-ST-segment elevation myocardial infarction (NSTEMI). Individuals who develop CS as a result of NSTEMI undergo early cardiac catheterization to a lesser extent, hence increasing the threat of mortality compared to individuals who develop CS due to STEMI. There have been improvements in in-hospital mortality; however, a minimal change is observed in the six to 12-month mortality (around 50%) in CS over the past couple of decades [7-9].

Between 5% and 13% of patients with AMI develop CS (AMI-CS) [10]. Early revascularization is vital in AMI-CS patients. Revascularization can be done within 18 hours of CS diagnosis. The most effective treatment to regain coronary flow in the affected coronary artery is primary percutaneous coronary intervention (PCI). The novel left ventricular unloading devices enable efficient hemodynamic support and help maintain perfusion to essential organs such as the brain, bowel, and kidneys [11].

## Review

Mechanical assist devices

The use of mechanical support devices is pivotal in managing CS. For many years, intra-aortic balloon counterpulsation was the conventional treatment used in patients with AMI-CS. However, after the disappointing outcomes of the IABPSHOCK II (Intraaortic Balloon Support for Myocardial Infarction with Cardiogenic Shock) study, the usage of intra-aortic balloon pumps (IABP) saw a decline, especially in Europe [8,12-14]. This yielded the rising use of active mechanical tools such as venoarterial extracorporeal membrane oxygenation (ECMO) and microaxial left ventricular assist devices (Impella, Abiomed, Danvers, MA) [8,14,15]. Although, there is currently a dearth of solid evidence encouraging the effectiveness of these devices.

Mechanical assist devices facilitate mechanical circulatory support (MCS) by unloading the ventricle, which lowers the cardiac filling pressures, pulmonary congestion, myocardial oxygen consumption, and wall stress. This allows time for myocardial recovery or time to decide if they might benefit from a longer usage of a ventricular assist device (VAD), either as a bridge to transplantation or as a destination therapy [16]. There are four types of circuit configurations for percutaneous MCS: (1) devices that pump the blood from the left ventricle (LV) to the aorta (IABP and Impella); (2) devices that assist in the left atrium (LA) to systemic artery circulation (TandemHeart, Cardiac Assist, Pittsburgh, PA); (3) devices that assist in the right atrium (RA) to systemic artery circulation (venoarterial extracorporeal membrane oxygenation (VA-ECMO)); and (4) devices that pump the blood from RA to pulmonary artery (adapted TandemHeart and Impella RP). All the devices that are currently available result in the improvement of blood pressure and cardiac output, but owing to their distinctive features, they result in different hemodynamics [1]. There is a lack of clarity on the effects of the disparities on clinical results. In this article, we review the current evidence and science behind Impella and ECMO devices in CS.

Impella

Percutaneous catheter-based transvalvular devices used with regard to short-term usage are devices that pump blood to the arterial system from the LV. Impella CP, Impella 2.5, Impella 5.0, Impella 5.5, and Impella LD families (Abiomed, Danvers, MA) of devices are examples of percutaneous transvalvular devices and they can be implanted by a minimally invasive procedure [17].

**Figure 1:**
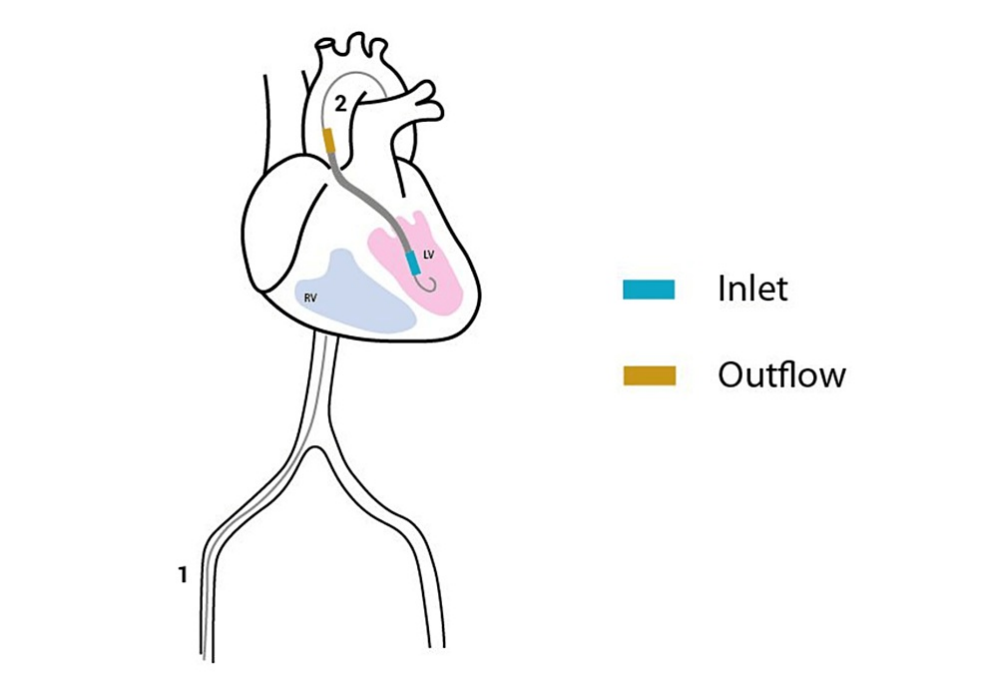
Percutaneous insertion of Impella catheter via the femoral artery. Inlet of the Impella is from the left ventricle and the outlet is in the ascending aorta. 1: femoral artery; 2: ascending aorta; RV: right ventricle; LV: left ventricle. Image credit: Neel Vora.

It gives patients temporary MCS, which reduces myocardial stress and enhances systemic circulation. Devices like Impella can be used during high-risk percutaneous coronary intervention (HRPCI) in patients diagnosed with cardiomyopathy and critical myocarditis or they can also be used for the treatment of AMI-CS [18,19]. Impella devices are transplanted across the aortic valve into the LV and they deliver the blood flow to the aorta from the LV (Figure 1). This not only reduces the LV work but also boosts the cardiac output resulting in improvement in systemic perfusion [18,19].

Impella is markedly the most commonly used transvalvular flow invention, with options for MCS both for left and right ventricles. The Impella CP, Impella RP, and Impella 5.0/5.5 are the devices that are currently offered. Impella 2.5, 5.0, 5.5, and CP are left-sided devices and the Impella RP is a right-sided device. Impella 2.5 and 5.0 enhance cardiac output up to 2.5 liters/minute and 5.0 liters/minute, respectively, and the Impella CP provides intermediary support of 3.0-4.0 liters/minute of cardiac output [20].

Impella could be a really beneficial device for patients with CS. Results of the ISAR-SHOCK trial showed that Impella quickly reversed serum lactate levels and offered various hemodynamic benefits [20].

Extracorporeal membrane oxygenation

Extracorporeal venoarterial membrane oxygenation, which is also known as extracorporeal life support (ECLS), has a pump to maintain the cardiac output as well as a gas exchange unit that maintains the normal oxygen and carbon dioxide partial pressures [17]. Venovenous extracorporeal membrane oxygenation (VV-ECMO) is typically reserved for patients who suffer from isolated pulmonary disease; however, VA-ECMO gives cardiopulmonary support in CS patients, which acts as a buffer to achieve myocardial rehabilitation. For patients with CS, VA-ECMO has been a potential treatment approach. In contrast to Impella, which pumps blood from LV to the arterial system, ECMO provides right atrial to arterial circulatory support [21].

**Figure 2:**
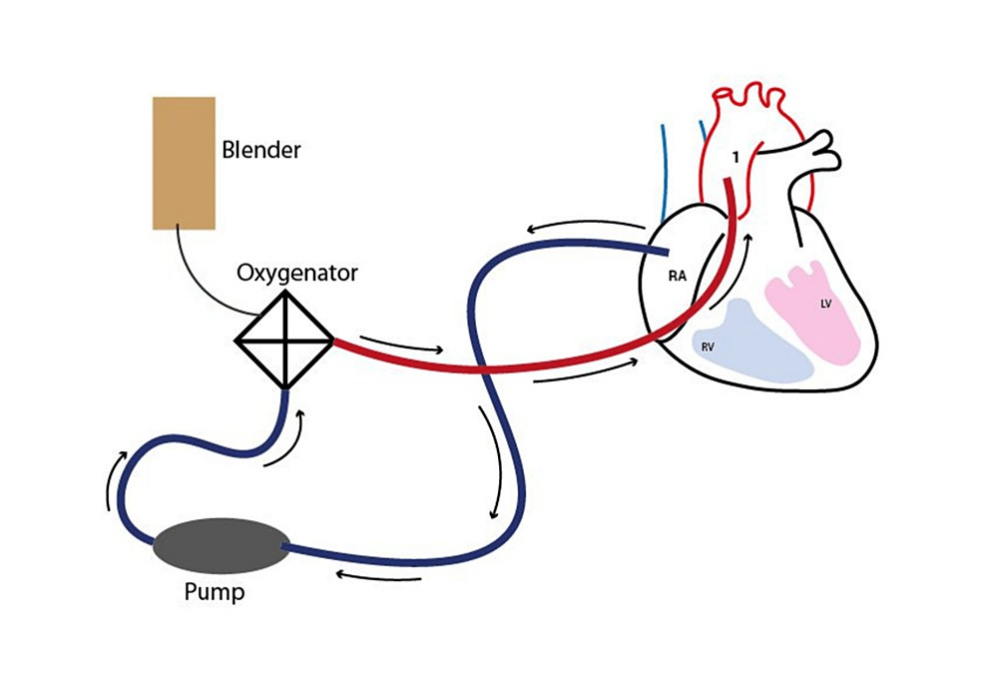
Central cannulation for venoarterial extracorporeal membrane oxygenation (VA-ECMO) support RA: right atrium; RV: right ventricle; LV: left ventricle; 1: ascending aorta. Image credit: Neel Vora.

**Figure 3:**
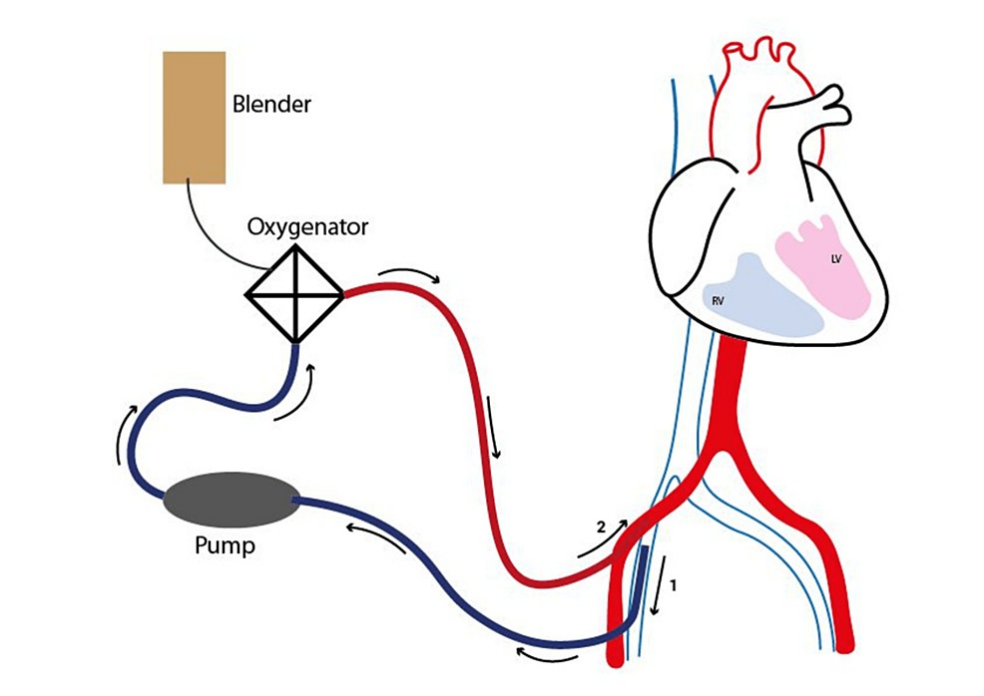
Peripheral cannulation for venoarterial extracorporeal membrane oxygenation (VA-ECMO) support. RV: right ventricle; LV: left ventricle; 1: femoral vein; 2: femoral artery. Image credit: Neel Vora.

VA-ECMO can be placed surgically using the central cannulation technique (Figure 2) where oxygenated blood is rendered to the ascending aorta; however, peripherally placed VA-ECMO (Figure 3) is a more often used configuration in patients having refractory CS. The femoral artery is approached and vein cannulation is done in percutaneous VA-ECMO. To avoid vascular injury by the cannula and to avoid significantly high outflow pressure and negative inflow, the selection of a cannula of proper diameter is essential [5,22]. Hybrid ECMO setups are becoming more common in lung injury patients or in those patients who are not adequately managed by VV-ECMO or VA-ECMO. One of the most widely employed techniques is the venoarteriovenous (V-AV) configuration. Deoxygenated venous blood is oxygenated via the oxygenator and later reinfused to the femoral artery via the arterial cannula and to the right heart by a venous cannula, hence providing highly oxygenated pulmonary blood circulation. This arrangement prevents the Harlequin (north/south) syndrome [23].

Full VA-ECMO support maintains normal hemodynamics in addition to gas exchange, which allows time for diagnostic and therapeutic interventions in addition to providing time for organ recovery [24,25]. Myocardial stroke work is increased in CS because of various vascular and neurohormonal mechanisms [17,26]. VA-ECMO is capable of reducing myocardial work and as a result, reducing pressure-volume area, which accounts for the reason for using VA-ECMO in CS [27,28].

Left ventricular end-diastolic volume (LVEDV) and left ventricular end-diastolic pressure (LVEDP) are significantly decreased with the use of the VA-ECMO as a result of a reduction in right ventricular preload and transpulmonary blood circulation [29-31]. The reduction of LVEDV and LVEDP promotes hemodynamic stabilization in CS and cardiac arrest.

ECMO is regrettably ineffective in severe left heart failure, particularly when used alone [32]. Due to a rise in diastolic pressure and an increase in ventricular expansion, it might worsen LV function and result in pulmonary edema. To lessen the afterload and preload in these circumstances, Impella (and IABP) can be utilized as a supplemental treatment, respectively [33].

Complications of Impella

Regardless of the type of device adopted, the implantation of an assist device is a major interventional procedure, which might lead to undesired complications such as thromboembolism and bleeding, as evidenced by numerous studies [34].

According to a study done between 2006 and 2011, where 47 patients with CS were given an Impella left ventricular assist device (LVAD), 72% of the patients showed improvement in ventricular function, and then the device was withdrawn; however, 8% of the patients switched to the long-term VADs. The 30-day mortality rate was found to be 25%. Of the patients, 30% faced impediments such as groin hematoma, device malfunction, tube fracture, and high purge pressures among others [35]. This study highlights that usage of such devices comes not only with human complications, but also issues pertaining to the device itself, which could be a significant drawback.

Device-related complications can pose a major issue. A study assessing the outcomes of the Impella device in 16 patients having CS revealed various device-related issues, including three sensor failures, one pump displacement, and six instances of hemolysis [34].

Similarly, a meta-analysis of outcomes of Impella in CS imparts further insight regarding the impediments. The rates of vascular and bleeding complications were 7.4% and 15.6%, respectively, according to the findings. A higher incidence of these consequences was found in older patients and women, indicating the importance of peripheral arterial disease in older patients and the small size of arteries in women. Additionally, the findings indicate an increased risk of vascular complications when using Impella 5.0 [36]. The utilization of a large French size (23F) with Impella 5.0 is another significant factor. It may be linked to the increased prevalence of vascular problems and preferably suggests the usage of Impella CP where clinical circumstances permit. A prior meta-analysis [36] indicated a bleeding rate of 23.1% in contrast to this study. This disparity could possibly be explained by the fact that only significant bleeding incidents requiring considerable clinical interventions, such as surgery and blood transfusions, were recorded in the meta-analysis. Nonetheless, the findings of both studies are significant, indicating that a large number of patients could be prone to a diverse array of complications following the use of Impella.

The 30-day mortality in 671 patients from 11 studies was 54.6%, according to another recent meta-analysis. Major bleeding was a complication in 10 studies, with a combined value of 19.9%; hemolysis was reported in nine studies, with a proportion of 10.5%; limb ischemia in seven studies, with a proportion of 5.0%; and stroke was recorded in seven studies, with a proportion of 3.8% [37].

Patients using Impella support devices need to have their hemolysis constantly checked. In accordance with the findings of Esposito et al., plasma-free hemoglobin is a highly specific and sensitive marker for hemolysis in Impella-assisted patients. As a result, it should be a standard practice to check lab evidence for hemolysis (i.e., bilirubin, haptoglobin, and lactate dehydrogenase) every six to 12 hours. If necessary, the frequency should be changed [38]. Significant hemolysis puts patients at risk for kidney damage, therefore early identification could save the organ. Patients with substantial hemolysis should be checked more frequently, whilst those without such symptoms can have lab testing less frequently [39].

Even though there are numerous complications associated with Impella, data from several studies have shown that the complications associated with the use of Impella are far fewer than that with VA-ECMO. Karami et al. reported that Impella 5.0 had a 17% incidence of complications like limb ischemia, bleeding of the access, and infection, compared to 40% with VA-ECMO. In particular, Impella 5.0 had a 1.1% incidence of infection of the access site compared to 15.8% for VA-ECMO [40]. In addition, the results of other observational studies support those of Karami et al. For instance, compared to VA-ECMO, Impella 5.0 was linked with a significant decrease in the incidence of arterial thromboembolism and significantly lower utilization of blood products according to a retrospective study [41]. Lemor et al. showed that their Impella cohort had a lower prevalence of acute respiratory failure, vascular consequences, acute renal damage, and severe respiratory failure, in addition to shorter hospital stays and lower healthcare expenses [42]. All these findings provide a favorable argument for the use of Impella.

It has been demonstrated that the patient prognosis is influenced by the timing of the Impella device placement. O'Neill et al. [43] evaluated two groups of patients who received PCI after presenting with AMI-CS. One group received Impella 2.5 before PCI, whereas the other group received it after PCI. According to their findings, patients who got Impella 2.5 before PCI had increased endurance rates and full revascularization in contrast to those in the post-PCI group [43]. This finding was reinforced further by a study by Basir et al. [44], wherein they demonstrated that the early incorporation of the Impella device before providing PCI has been associated with full revascularization and a good survival rate. Early Impella implantation before administering vasopressors or inotropes, and before PCI, has clearly been shown to be independently linked with increased survival in patients [45].

Apart from these complications associated with Impella use, there are also several associated limitations that need to be addressed such as the limited availability of the devices, difficulty in transporting to a tertiary care hospital, limited duration of support, and the lack of respiratory support [46].

Complications of ECMO

Pulmonary Edema

Using this circuit configuration can significantly increase LV preload and, in some circumstances, result in pulmonary edema. Baseline CS is characterized by a high LVEDP, low-pressure production, low stroke volume (SV), and low ejection fraction. The main hemodynamic consequence of initiating ECMO and stepping up the flow rate from 1.5 to 3.0 to 4.5 l/min is an increase in LV afterload pressure. If total peripheral resistance (TPR) and LV contractility are stabilized, there is no other way for LV to counteract the high afterload except by the Starling mechanism, leading to the retention of blood in the LV. As a result, there is a rise in the PCWP, LA pressure, and LVEDP, and the pressure-volume loop (PVL) shifts rightward and upward and it gets narrower (due to a drop in native LV SV) and taller (due to the high afterload). This significantly raises myocardial oxygen demand, leading to deterioration in LV function, particularly in conditions of acute myocardial ischemia or infarction [17].

A variety of factors contribute to the complexity and variability of hemodynamic responses to ECMO among patients. In some individuals, chest X-ray findings of pulmonary edema or a high PCWP are an indication that mechanical LV unloading is necessary. Variability in patient responses may be explained by variable effects of ECMO on LV contractility and TPR. However, ECMO alone may not result in appreciable LV unloading, even in the context of rather considerable secondary effects [17].

Vascular Complications

Numerous other complications have been reported. Vascular complications have a prevalence of 20-30%, with limb ischemia being the most commonly reported complication. Other vascular complications included hyperemia and compartment syndrome [47,48]. Other vascular complications such as perforation of the wall of a vessel, dissection of a vessel, aneurysm, and thrombosis can occur. The use of anticoagulants in VA-ECMO increases the likelihood of hematoma formation. This can be managed conservatively most of the time but some complications may need urgent surgical repair of the vessel [49]. Ischemia of the ipsilateral lower extremity is one of the most serious complications of VA-ECMO. The risk for developing this complication is more in young patients, female patients, difficulty in accessing the vessel, peripheral artery disease in the patient, and use of a cannula with an incorrect diameter (larger) [47,50,51]. There was a 16.9% incidence of lower limb ischemia when an analysis of 20 studies was done, which included 1,866 patients who were on VA-ECMO support for cardiac arrest or CS. Of these patients, 10.3% had a possibility of compartment syndrome or required fasciotomy. Amputation of the lower limb was required in 4.7% of patients [52].

Neurological Complications

Numerous neurological complications, including ischemic and hemorrhagic strokes, paraplegia, seizures, peripheral neuropathy, and even death, have been linked to the use of ECMO. The incidence of all neurological complications combined could be as high as 13.3%. Duration of ECMO support, thrombosis, and microemboli within the cannula, renal failure, and imbalance between anticoagulants and procoagulants are some of the risk factors believed to be implicated in neurological complications [53].

The results of a recent study comparing Impella and ECMO in CS found that the incidence of ischemic cerebrovascular accidents and hemorrhagic cerebrovascular accidents could range from 3.3% to 17.6% and 1.6% to 5%, respectively. This is significantly higher compared to the rates of Impella, which could range from 2.4% to 6.3% [54].

Infections

Bloodstream, lower respiratory tract, and urinary tract infections have an incidence of 2.98-20.55 episodes, 24.4 episodes, and 1-13.8 cases per 1,000 ECMO days, respectively [55]. The overall presence of infection is between 9% and 65% [56-59]. Scenarios, where emergency cannulation needs to be done, can be associated with difficulties in maintaining a sterile field, which can cause infection at the access site. A prolonged indwelling catheter is a source of infection from the urinary tract [59-61]. *Staphylococcus aureus*, *Candida*, and non-lactose fermenting gram-negative bacilli are common pathogens causing infection [58,60].

Bleeding Complications

The prevalence of bleeding complications is between 30% and 56%. The gastrointestinal tract, thorax, and cannula sites are typical sites. Bleeding is implicated to occur due to fibrinolysis, platelet defects, and elimination of von Willebrand factor multimers, which have all been linked to the use of ECMO. Bleeding is predicted by the HAT (hypertension, age > 65 years, and VA-ECMO type) score. A greater score suggests escalating transfusion needs [62-67]. Bleeding can also occur in the pericardium, cranium, retroperitoneal, and intraperitoneal areas. The factors responsible for bleeding may be blood exposure to artificial surfaces of the MCS system, anticoagulants used to avoid thrombus formation, platelet activation, increased activity of the fibrinolytic system, systemic inflammation, infection and sepsis with prolonged use of VA-ECMO, and trauma [68].

Renal Injury

Of the patients on the VA-ECMO, 55.6% are at threat of developing acute renal failure [69]. Acute kidney injury occurs as a result of an inflammatory response of the body, hemoglobinuria in case of hemolysis, embolism in renal vessels, and hypoperfusion of kidneys, which can result from an abnormality in the renin-angiotensin-aldosterone system [69-73]. According to a study, the most often experienced adverse events were renal failure and bleeding (50.5% and 48.5%, respectively). Of the patients, 44.3% required renal replacement therapy [74].

Systemic Inflammatory Response Syndrome

Another complication is systemic inflammatory response syndrome (SIRS). Most ECMO patients experience some level of systemic inflammation, and 30% of patients experience it after decannulation [75,76]. The higher incidence of various complications such as bleeding and vascular complications in ECMO-assisted patients is likely a result of the use of larger cannulas than that needed for Impella [42].

North-South (Harlequin) Syndrome

North-South (Harlequin) syndrome may occur upon the use of peripheral VA-ECMO when there is a recovery in the cardiac function but not in the pulmonary function, hence deoxygenated blood from the lungs travels to the LV, which is then pumped to the systemic circulation. This leads to the mixing of the anterograde flow of the deoxygenated blood and the retrograde flow of the fully oxygenated blood provided by the circuit [77]. In the presence of aberrant pulmonary gas exchange, blood infusing the heart, upper limbs, and brain might have a sub-90% oxygen saturation, even when mixed with blood from the femoral arterial cannula, which is adequately oxygenated, leading to upper body cyanosis [78,79].

Recent research, however, has also shown that the percutaneous cannulation for peripheral VA-ECMO is correlated with the reduction in the incidence of infections and enhanced 30-day survival when weighed up to the surgical approach, suggesting that this method used to deliver each MCS therapy too shall have an impact on results [80].

Mortality rate

Results with the use of VA-ECMO for CS have been underwhelming. Patients on VA-ECMO assistance continue to have a poor chance of survival despite technological advancements, with a 50-60% in-hospital mortality and six-month survival of around 30% [81-83]. Studies reveal that survival rates after five years are only about 25% regardless of whether ECMO was prescribed for cardiac failure or respiratory failure. ECMO is a band-aid that is often only applied for a few days in patients with CS. If more time is required for support, an Impella should be implanted in the axilla as a stopgap measure until a transplant, recovery, or durable LVAD can be used [42]. A meta-analysis of 32 studies conducted between 1994 and 2019 was done, which included 12,756 patients. An average of 5.3 days was the duration of ECMO support. The in-hospital death rate was 62%. The number of patients who died during ECMO support was more than one-third (37.4% of all patients). The percentage of patients who experienced cardiac arrest before or during ECMO implantation was 30.2% [74]. These findings can be accredited to the higher incidence of complications and adverse effects related to the use of ECMO.

Most of the studies have shown that there is not much variation in mortality of patients treated with either Impella or ECMO, while some argue that the in-hospital death rates are higher in ECMO than in Impella [42]. It was discovered that the in-hospital mortality was considerably greater for the ECMO cohort in a recent study comparing the results of Impella and ECMO (45.5% vs. 41.4%). Cardiopulmonary resuscitation (CPR) rates (19.5% vs. 31.3%) and rates of cardiac arrest (17.5% vs. 25.9%) were both considerably greater in the ECMO cohort [42]. However, the analysis of a different study showed no significant difference in 30-day (43.3% vs. 58.1%) and six-month (48.5% vs. 64.5%) mortality rates in VA-ECMO and Impella-treated patients [84]. This finding was proven by yet another study, which did not show any significant difference in the 30-day mortality between the ECMO and Impella groups [41]. Additionally, another study comparing the survival of CS patients with Impella and ECMO showed no difference in the survival among patients in the intensive care unit (65% for the ECMO patients and 63% for Impella patients), nor the long-term survival amongst the two groups [85]. These findings, however, do reinforce the fact that while there may be a negligible difference in the mortality of patients managed with either Impella or ECMO, there is a greater chance of in-hospital mortality rates with ECMO than with Impella.

Findings have clearly been in favor of Impella. A Canadian study [86] found that even though the patients assisted with Impella had a high in-hospital mortality, there was a comparatively modest expansion in mortality at one year. This implies that individuals in CS who are treated with Impella are likely to still be alive a year later provided they survive their hospital stay. This result was previously demonstrated in patients undergoing IABP [87] and other forms of MCS, such as ECMO [88]. They also discovered that the number of Impella patients who ultimately underwent cardiac transplantation was substantially lower than the number of patients who received ECMO [88].

Length of hospital stay and costs

Another important aspect to focus on when comparing Impella and ECMO is the length of hospital stay and the costs corresponding to both devices. A study by Lemor et al. showed that hospital costs were lower and hospital stays were shorter in patients assisted with Impella when compared to ECMO [42].

Impella use is linked to a 52% decrease in the number of times a patient is admitted for revascularization at 90 days [89], a reduction in the duration of hospital stay [90], and a reduction in the risk for acute kidney injury, with around $22,023 cost savings per case [91]. In both Europe and the United States, these results are constant. Analysis using the USpella and Europella databases has also found Impella to be a more affordable treatment option for high-risk PCI patients than IABP [92]. Indeed, the use of the Impella has been linked to better outcomes, a shorter duration of hospital stay, fewer problems, and higher cost savings as compared to ECMO [42].

One of the most conservative regulatory organizations in the world, the National Institute for Health and Care Excellence (NICE), approved the use of Impella in some high-risk PCI patients as evidence of its cost-effectiveness. Incremental cost-effectiveness ratio (ICER) is a common economic term that measures how much more expensive a healthcare intervention is relative to the next best option for various healthcare outcomes, such as a quality-adjusted life year. Impella's ICER reveals a $135,000 annual cost savings in an emerging population [93].

The cost of Impella 5.0 and VA-ECMO was also compared using a budget impact model in a French study. The total expenditures for patients with CS without the implementation of Impella 5.0 were estimated to be 90.8 million euros, including the direct costs attributed to various complications. However, with the implementation of Impella 5.0, the costs were expected to be 86.5 million euros over five years. Therefore, it was predicted that if Impella 5.0 is implemented in clinical practice, there will be a savings of 4.3 million euros over the course of five years. The total yearly savings were anticipated to rise constantly as the market share of Impella increased. The reduction in the number of complications and decreased duration of stay with Impella 5.0 were the main factors driving the predicted savings linked with the implementation of Impella 5.0 [94].

The same group also compared the economic implications of using the Impella CP to VA-ECMO and IABP in patients with CS in France. According to their estimates, implementing Impella CP would result in cost savings of 2.7 million euros over a five-year time frame compared to maintaining the present clinical practice with VA-ECMO and IABP alone [95].

According to a different study, their Impella cohort experienced considerably shorter hospital stays (seven days vs. 11 days) and reduced expenses ($66,078 vs. $122,996) than the ECMO group [65]. ECMO-assisted patients had average hospital costs of around $580,066 per patient, according to a different US-based analysis, as opposed to $156,437 per patient without ECMO [96]. The average cost per visit for CS assisted with ECMO is greater now than it was between 1998 and 2009 when it was $344,009 (±$30,707) [97]. Costs related to ECMO are therefore increasing. These outcomes strongly support the favorable economics associated with the widespread implementation of Impella, which can reduce the patient burden exponentially.

However, it should be kept in mind that the usage of Impella devices is not cheap. It is a comparatively cheaper alternative to ECMO, which makes it a viable treatment option in patients with CS. A Canadian study evaluated the costs in the first year after Impella in 110 patients. Total expenses were $88,397 on average [87]. The expense of using Impella was high and equivalent to but still less than the cost of using an ECMO in Ontario [88].

It is crucial to remember that even while using Impella may result in cost savings, there are some situations in which using VA-ECMO might be a better course of action. For patients having refractory cardiac arrest or respiratory or multiorgan failure, VA-ECMO is the preferred management option [98].

## Conclusions

Impella and VA-ECMO differ significantly in several ways that could have an impact on clinical judgment. For some CS patients, for instance, a major benefit of using Impella is the direct unloading of the LV. VA-ECMO, on the other hand, oxygenates the blood and provides circulatory support without emptying the LV. In conclusion, this may increase the LV pressure, which may then stress the myocardium. In turn, VA-ECMO therapy has been linked with a worse outcome in CS patients with LV failure. In the context of AMI, ventricular unloading has lately come to be recognized as a critical technique for limiting ischemia-reperfusion injury, promoting myocardial healing, and minimizing infarct size.

While several studies have shown that Impella use is linked to better outcomes, lower mortality rates, and fewer complications as compared to VA-ECMO, there is still a lot of scope for availing more data to better evaluate Impella and VA-ECMO in CS patients. There are data available that suggest pairing Impella with ECMO provides the optimum hemodynamic support; however, more studies are required to evaluate the same. Several ongoing trials, such as the REVERSE (NCT03431467) and UNLOAD ECMO (NCT05577195) trials, will provide valuable insights regarding the utilization of Impella and ECMO in patients with CS.
